# Medication Adherence in Type 2 Diabetes: The ENTRED Study 2007, a French Population-Based Study

**DOI:** 10.1371/journal.pone.0032412

**Published:** 2012-03-05

**Authors:** Michel Tiv, Jean-François Viel, Frédéric Mauny, Eveline Eschwège, Alain Weill, Cécile Fournier, Anne Fagot-Campagna, Alfred Penfornis

**Affiliations:** 1 Medical Information Department, University Hospital of Besançon, Besançon, France; 2 Institut National de la Santé et de la Recherche Médicale (Inserm), Villejuif, France; 3 French National Health Insurance Fund for Salaried Workers (CnamTS), Paris, France; 4 Institute for Health Prevention and Education (Inpes), Saint Denis, France; 5 Institute of Public Health Surveillance (InVS), Saint-Maurice, France; 6 Department of Endocrinology-Metabolism and Diabetology-Nutrition, Jean Minjoz Hospital, University of Franche-Comté, Besançon, France; Universidad Peruana Cayetano Heredia, Peru

## Abstract

**Background:**

Adherence to prescribed medications is a key dimension of healthcare quality. The aim of this large population-based study was to evaluate self-reported medication adherence and to identify factors linked with poor adherence in patients with type 2 diabetes in France.

**Methodology:**

The ENTRED study 2007, a French national survey of people treated for diabetes, was based on a representative sample of patients who claimed reimbursement for oral hypoglycaemic agents and/or insulin at least three times between August 2006 and July 2007, and who were randomly selected from the database of the two main National Health Insurance Systems. Medication adherence was determined using a six-item self-administered questionnaire. A multinomial polychotomous logistic regression model was used to identify factors associated with medication adherence in the 3,637 persons with type 2 diabetes.

**Principal Findings:**

Thirty nine percent of patients reported good medication adherence, 49% medium adherence and 12% poor adherence. The factors significantly associated with poor adherence in multivariate analysis were socio-demographic factors: age <45 years, non-European geographical origin, financial difficulties and being professionally active; disease and therapy-related factors: HbA_1c_>8% and existing diabetes complications; and health care-related factors: difficulties for taking medication alone, decision making by the patient only, poor acceptability of medical recommendations, lack of family or social support, need for information on treatment, reporting no confidence in the future, need for medical support and follow-up by a specialist physician.

**Conclusions:**

In a country with a high level of access to healthcare, our study demonstrated a substantial low level of medication adherence in type 2 diabetic patients. Better identification of those with poor adherence and individualised suitable recommendations remain essential for better healthcare management.

## Introduction

Diabetes mellitus, a complex chronic disease, is a growing worldwide epidemic with the number of people with diabetes estimated to reach 330 million by 2030 [1]. Given the high morbidity and mortality associated with the disease, primarily due to macrovascular complications, type 2 diabetes is a major public-health concern. Most European countries have formulated evidence-based guidelines with clear targets [2], [3], but actual care often falls far short of these targets [4], [5].

A key dimension of healthcare quality is adherence to prescribed medications. According to the World Health Organization (WHO), adherence is the extent to which a person's behaviour – taking medication, following a diet, and/or executing lifestyle changes – corresponds with agreed recommendations from the health care provider [6]. However, medication non-adherence is particularly common among patients with type 2 diabetes [7] and inadequate adherence compromises safety and treatment effectiveness, leading to increased mortality and morbidity with considerable direct and indirect costs to the healthcare system [8], [9]. A recent WHO report states that, because the magnitude of non-adherence and the scope of its sequelae are so alarming, more health benefits worldwide would result from improving adherence to existing treatments than by developing new medical treatments [6].

Previously, numerous studies have explored potential risk factors of adherence to medicines across a variety of conditions. However, the majority of studies have explored largely unmodifiable variables due to the retrospective databases that are often used to measure adherence. Frequently cited risk factors include age, sex, ethnicity, income, education, and comorbidity though their relationship to adherence has been inconsistent due to variations in study designs and sample populations [7], [10]–[12]. Therefore there is a continuing need to better identify factors related to medication adherence. Moreover, previous studies on medication adherence often included a limited number of diabetic patients and with selected patient population [13]–[27], which limited the generalizability of the results. This large population based study, the largest European study to our knowledge, was conducted to evaluate medication adherence in people with type 2 diabetes, and to identify the risk factors for poor adherence and especially, modifiable factors.

## Methods

### Ethics Statement

The French National Ethics Committee and the French Data Protection Authority Committee gave its approval to the ENTRED study. According to the French regulations, written consent for filling questionnaire was not required as no intervention was performed on the participants and no blood or human tissue was considered. Filling a self-reported questionnaire and mailing it back was thus considered as consent for all participants. For medical data filled by medical practitioners, participants who provided addresses of their care practitioners filled a form which was sent to the care practitioners to authorize them to provide further medical information.

### Study population and design

The ENTRED study was a French national public survey. A complex random sample was selected from all patients aged over 18 years who claimed reimbursement for oral hypoglycaemic agents (OHA) and/or insulin at least three times between August 2006 and July 2007, from the two main National Health Insurance Systems (NHIS), which cover all active and retired employees and their relatives—about 80% of the French population. To classify the different types of diabetes, we used an epidemiological algorithm: people diagnosed before the age of 45 years and treated with insulin within two years from diagnosis were classified as having type 1 diabetes and have not been considered for this study.

A detailed questionnaire with a total of 110 questions was sent to all patients (48% response rate; n = 3,973 with 3,637 type 2 diabetic patients) and a medical questionnaire was also sent to the medical-care providers of those among the responders who gave their medical provider's addresses (63% response rate; n = 2,485). Care providers reported the most recent clinical measurements.

Medication adherence, the dependant variable, was analysed in the subgroup of people with type 2 diabetes. The independent variables to explain the medication adherence included socio-demographic characteristics (age, gender, education level, geographical origin, marital status, residence, professional activity, financial level, complementary health insurance…), characteristics associated with disease and therapy (time since diabetes diagnosis, type of treatment, body mass index, hypertension, dyslipidaemia, smoking state, glycaemic control, microvascular or macrovascular complications…), and associated with medical care (decision making, follow-up by a specialist, acceptability of medical recommendations, ability for taking medicine alone, need for medical support or information on treatment…).

### Measure of medication adherence

In this study, medication adherence (referring to any medicine, not just for diabetes) was determined using a six item self-administered questionnaire, drawing upon the works by Girerd et al. [28]. Patients responded yes or no to each of the following questions: (1) do you sometimes forget to take your medicine, (2) have you ever run out of your medicine, (3) do you sometimes take your medicine late, (4) do you sometimes decide not to take your medicine because someday you feel that your treatment do more harm than good, (5) do you think that you have too many pills to take, (6) when you feel better, do you sometimes stop taking your medicine. It has been shown that such a questionnaire has sufficient validity and reliability [28]. Compared to a clinical evaluation of medication adherence, the values of kappa indices were 0.65 in “good adherence” when “No” was answered to the 6 items, 0.5 in “medium adherence” when 1 or 2 “Yes” were given and 0.56 in “poor adherence” when 3 or more “Yes” were given [28].

### Statistical analyses

Descriptive analyses were first performed. In data reported by patients, missing data for medication adherence were excluded from analyses. To minimise potential non-response biases, all analyses were weighted to take into account the participation rate based on socio-demographic data and the type of antidiabetic treatment. Quantitative values are expressed as means ± standard deviation, and were compared by Student's *t* test, analysis of variance or nonparametric test when appropriate, while qualitative values were compared by the χ^2^ test or Fisher's exact test. The outcome of interest, medication adherence, was classified into three categories; ‘good’, ‘medium’ and ‘poor’ and was treated as a nominal variable since the proportional odds assumption was rejected. A multinomial polychotomous logistic regression model was used to estimate the effect of each covariate on the odds of poor adherence and on the odds of medium adherence versus good adherence, while simultaneously adjusting for all other variables in the model. All statistical analyses took into account the sample survey design and were carried out using SAS version 9.1.3 (SAS Institute Inc, Cary, NC). The characteristics of those who responded to the detailed questionnaire were compared with those who did not, using the 2007 administrative data available for all people.

## Results

### Participants' characteristics

This study included a total of 3,637 type 2 diabetic patients (2,138 men and 1,499 women) with a mean age of 65 years (18 to 102 years). The main socio-demographic and clinical baseline characteristics of the responders are summarized in Table 1. Eighty one percent were treated with OHA without insulin, mean HbA_1c_ was 7.0% and 41% had microvascular or macrovascular complications. Respondents to the survey were slightly younger than non-respondents (64 years versus 66 years on average; p<0.0001), most frequently male (59% versus 52%; p<0.0001), most frequently born in France (79% versus 70%; p<0.0001), less likely to be treated with OHA without insulin (74% versus 78%; p<0.0001) and had better medical follow-up (43% versus 35% had three HbA_1c_ tests per year; p<0.0001).

**Table 1:** Main socio-demographic and clinical baseline characteristics of the 3,637 responders, Entred study.

Variables	Study sample
Age [SD]	65.0 [11.1]
Sex - n (%)	
male	2138 (54.4)
female	1499 (45.6)
Geographical origin - n (%)	
France	2694 (79.0)
Europe (except France)	373 (11.2)
other	346 (9.8)
Education level - n (%)	
low	529 (16.3)
medium	2177 (63.9)
high	769 (19.8)
Marital status - n (%)	
unmarried	1105 (32.8)
married	2491 (67.2)
Complementary health insurance - n (%)	
yes	3157 (88.0)
no	432 (12.0)
Financial difficulties - n (%)	
no	1668 (46.5)
few	1095 (31.8)
yes	762 (21.7)
Professional activity - n (%)	
yes	2929 (83.0)
no	672 (17.0)
Treatment - n (%)	
OHA	2859 (80.5)
insulin +/− OHA	742 (19.5)
Body mass index - n (%)	
<25 kg/m2	650 (19.0)
25–29 kg/m2	1405 (39.4)
≥30 kg/m2	1446 (41.6)
Smoking - n (%)	
yes	659 (17.4)
no	2950 (82.6)
Presence of complication - n (%)	
yes	1405 (41.4)
no	2014 (58.6)

SD: standard deviation; OHA: oral hypoglycaemic agents.

### Medication adherence

Thirty nine percent of patients had good adherence, 49% medium adherence and 12% poor adherence: 18% of patients reported sometimes forgetting to take their medicine, 9% running out of their medicine, 38% sometimes taking their medicine late, 4% sometimes deciding not to take their medicine because someday they felt that their treatment do more harm than good, 34% having too many pills to take and 5% sometimes stopping to take their medicine when they felt better.

In univariate analysis, many factors were associated with medication adherence (not detailed). On the contrary gender and duration since diagnosis did not affect medication adherence (p = 0.93 and 0.90, respectively). Patients with professional activity (who currently work) forgot more often to take their medicine (30% versus 15%, p<0.0001) and took more often their medicine late (51% versus 35%, p<0.0001).

In polychotomous logistic regression (Table 2), socio-demographic factors significantly associated with poor (versus good) adherence were: age <45 years (Odds Ratio (OR) = 5.2), non-European geographical origin (OR = 2.6), financial difficulties (OR = 1.7) and being professionally active (OR = 1.5). Disease and therapy-related factors significantly associated with poor adherence were: HbA_1c_>8% (OR = 2.0) and existing diabetes complications (OR = 1.7). A trend was observed with self-reported hypertension (OR = 1.4, p = 0.08) and dyslipidemia (OR = 1.4, p = 0.08) while being treated with insulin and diabetes duration did not influence medication adherence. Lastly, health care-related factors significantly associated with poor adherence were: difficulties for taking medication alone (OR = 3.8), decision making by the patient only (OR = 3.3), poor acceptability of medical recommendations (OR = 2.7), lack of family or social support (OR = 2.5), need for information on treatment (OR = 2.0), reporting no confidence in the future (OR = 1.6), need for medical support (OR = 1.6) and follow-up by a specialist physician (OR = 1.4).

**Table 2:** Multivariate analysis of medication adherence. Entred study, N = 3637.

			Good adherence	Medium versus good adherence			Poor versus good adherence		
Variables	Subcategory	P-value	N	N	OR	95% CI	N	OR	95% CI
**Socio-demographic factors**									
Age (years)	18–44	**<0.0001**	23	50	1.8	1.1–3.1	38	5.2	2.7–10.1
	45–64		522	888	1.5	1.2–1.8	236	1.8	1.3–2.4
	65–84		808	815	1		147	1	
	85 and more		63	37	0.5	0.3–0.8	10	1.0	0.5–2.0
Geographical origin	France	**<0.0001**	1,085	1,340	1		269	1	
	Europe*		138	182	1.0	0.8–1.3	53	1.3	0.9–1.9
	other		87	172	1.3	1.0–1.8	87	2.6	1.8–3.9
Complementary health insurance	no	0.12	167	192	1		79	1	
	yes		1,229	1,581	1.3	1.0–1.6	347	0.9	0.6–1.3
Financial difficulties	no	**0.02**	739	780	1		149	1	
	few		417	559	1.2	1.0–1.4	119	1.1	0.8–1.5
	yes		203	408	1.4	1.1–1.7	151	1.7	1.2–2.4
Professional activity	no	**0.04**	1,199	1,423	1		307	1	
	yes		200	356	1.2	0.9–1.5	116	1.5	1.1–2.1
**Disease and therapy-related factors**									
Treatment	OHA	0.15	1,158	1,377	1		324	1	
	insulin +/− OHA		248	393	1.1	0.9–1.3	101	0.9	0.6–1.2
Glycated haemoglobin	≤6.5%	**0.01**	235	234	1		36	1	1
	6.5–8%		260	355	1.4	1.1–1.8	65	1.7	1.1–2.7
	>8%		66	105	1.4	1.1–1.7	31	2.0	1.3–3.0
Presence of microvascular or macrovascular complications	no	**0.0009**	834	970	1		210	1	
	yes		487	723	1.3	1.1–1.6	195	1.7	1.3–2.2
Hypertension	no	0.08	580	623	1		144	1	
	yes		778	1,099	1.2	1.0–1.4	269	1.4	1.0–1.8
Dyslipidemia	no	0.08	627	690	1		144	1	
	yes		700	1,014	1.1	1.0–1.3	267	1.4	1.1–1.8
**Health care-related factors**									
Difficulties for taking medicine alone	no	**0.0002**	1,326	1,660	1		373	1	
	yes		28	80	1.8	1.1–2.8	41	3.8	2.2–6.9
Decision making	physician and patient	**<0.0001**	854	909	1		191	1	
	physician		411	637	1.4	1.1–1.6	146	1.5	1.2–2.0
	patient		73	168	1.9	1.4–2.6	63	3.3	2.1–5.0
Acceptability of medical recommendations	good	**0.004**	1,174	1,440	1		307	1	
	poor		30	123	2.1	1.4–3.3	56	2.7	1.6–4.6
Family or social support	yes	**0.0002**	1,064	1,302	1		271	1	
	no		88	224	1.4	1.0–1.9	102	2.5	1.7–3.6
Need for information on treatment	no	**<0.0001**	1,162	1,368	1		303	1	
	yes		137	327	1.7	1.3–2.1	106	2.0	1.4–2.7
Confidence in the future	yes	**0.0009**	1,040	1,091	1		199	1	
	no		275	613	1.5	1.2–1.8	203	1.6	1.2–2.2
Need for medical support	no	**0.002**	1,045	1,122	1		214	1	
	yes		371	668	1.2	1.0–1.5	217	1.6	1.2–2.1
Follow-up by a specialist	no	**0.005**	1,271	1,560	1		352	1	
	yes		145	230	1.1	0.9–1.4	79	1.4	1.2–2.5

* Except France; OR: odds ratio; 95% CI: 95% confidence interval; OHA: oral hypoglycaemic agents.

## Discussion

Medication adherence is a key component of self-management for patients with diabetes. Our population-based study found a low rate of good adherence (39%), which means that for many patients, medication adherence could be improved. One of the most common challenges physicians face with a patient with poorly controlled diabetes is to try to figure out if the patient's hyperglycemia is due to poor adherence or is occurring despite proper medication use (i.e., therapy needs to be intensified). Since patients may be more willing to report suboptimal adherence (self-reports typically provide overestimates of adherence for several reasons: first, they may rely on patients' own interpretation or memory of what advice was given and, if accepted, how closely it has been followed; second, patients may tend to report higher levels of adherence in order to please health care providers or avoid embarrassment), probing the handful of strongly predictive factors we have identified is useful for two reasons. First, it can help identify those likely to be poor adherers. Second, it can direct the physician on aspects of diabetes and its management on which they should focus their patient education efforts [29].

Our results are consistent with previous studies and particularly, with the DARTS study which found that adequate adherence (adherence index ≥90%) was found in only approximately one in three of those with type 2 diabetes receiving OHA [16]. Nonetheless, a recent meta-analysis found that medication adherence ranged from 36% to 93% depending on the definition applied [7]. However, the lack of standard measurements prevents comparison being made between studies and across populations. We also found that poor glycaemia control and presence of microvascular or macrovascular complications were more common among patients with poor adherence to medications.

In previous studies, many factors were inconsistently found as risk factors of poor adherence to drug therapy in type 2 diabetes. The statistical power of this study based on a large sample size was sufficient to detect small differences. Age [16], [30], financial difficulties [6], ethnicity [23], [24], psychological factors [31], social support [6], quality of the relationship between patient and physician [32] were confirmed as risk factors of medication adherence. In this study, we particularly focused on modifiable factors associated with medical care (decision making, acceptability of medical recommendations, medical support …). Contrary to Lawton et al. who found that non-adherence was related more to patient forgetfulness than to specific concerns about medications or interaction with physicians [33], our results show the importance of shared decision-making in which the beliefs and preferences of the patient are taken into consideration. This patient centred approach should enhance adherence and improve outcomes [34], [35]. Other interesting risk factors of poor adherence found in this study were the presence of difficulties for taking medicine alone, need for information and poor acceptability of medical recommendations. These two last findings confirm the need to better inform patients about their disease and treatment and to individually adapt recommendations. This could underline the insufficiency of patient education especially since it is now well established that education enables the patient to acquire knowledge and understanding of diabetes, self-management skills and psychosocial competencies [36], [37]. Social or family support is also crucial. Family members are frequently involved and recognized as supportive: they act as counsellors encouraging diet and exercise behaviours, facilitating adherence with medication, and altogether helping patients to “live with the disease” [38]. Lastly, the fact that professional activity was associated with poor adherence could be surprising considering a potential healthy worker effect. However, we clearly report that active patients more often forget to take their medicine and/or take their medicine late, and the relationship remains significant after adjustments.

Ultimately, two categories of risk factors for poor adherence could be distinguished: unmodifiable factors (such as age, ethnicity…) or factors that are hardly modifiable in the context of the medical relationship (financial difficulties, presence of professional activity…) which may help physicians to better identify patients at high risk for poor adherence and to adapt their medical care ; and some modifiable factors (such as social support, quality of the relationship between patient and physician, need for information, poor acceptability of medical recommendations…) on which physicians could focus their efforts to improve medication adherence and, as a consequence, to improve glycaemia control. In our study, no significant difference in adherence was found between males and females, which has already been shown [16]. However, it should be noted that the DIABASIS study evidenced clear gender differences in the perception and self-management of disease. Women took the disease more seriously, reported a higher impact on daily life and were more involved in self-management, while men relied more on family support. The authors suggested that physicians should take these differences in attitudes into account when counseling, educating and treating patients [38].

After identifying patients at high risk for poor adherence, the physician should try more than usual to apply multiple interventions in order to improve adherence: educational, behavioral, and affective interventions [39]. Educational interventions seek to improve adherence by providing information and/or skills. Education may take the form of individual instruction or group classes. In any event, a key element of successful educational strategies is providing simple, clear messages, hopefully tailored to the needs of the individual, and verifying that the messages have been understood. Behavioral approaches have their roots in cognitive-behavioral psychology and use techniques such as reminders, memory aids, synchronizing therapeutic activities with routine life events (e.g., taking pills before you shower), goal-setting, self-monitoring, contracting, skill-building, and rewards. For example, reminders may be mailed, e-mailed, or telephoned. What is important is that the behavior in question has been negotiated with and accepted by individual patients so that adoption of the behavior has a chance of succeeding in the long term. Affective interventions seek to enhance adherence by providing emotional support and encouragement. Finally, it should be remembered that application of multiple interventions of different types is more effective than any single intervention [39].

Our results should be viewed with consideration of several limitations. One limitation was the use of self-report data on medication adherence, because of a resulting tendency to overestimate adherence due to recall biases and social desirability. However, self-reported questionnaires have frequently been used because they are low in both cost and time expenditure and appropriate for large population-based samples. Subsequent research suggests that the self-report methods provide a reasonably accurate estimate of adherence [40]. Besides, our results based on self-report questionnaires were consistent with the literature, poor glycaemic control being more common among patients with low adherence to medications [41], [42] and many well-known factors associated with poor adherence were also identified by our study. The total number of medications prescribed to the patient has not been assessed in our study. However, this factor has been recognized as a contributor of patient adherence for a long time [43] and does not need any more to be established. Biases linked to participation are a common limitation, although our response rate was in keeping with population-based surveys. It is possible that respondents were more concerned about their diabetes than the others and, as a result, this may have led to an overestimation of medication adherence. To account for potential non response biases, we weighted our results according to the participation rate, based on socio-demographic data and type of antidiabetic treatment, as these auxiliary variables were correlated with both the non-response process and the survey estimate [44], [45]. Lastly, our study was cross sectional, where causal relationship between the independent and dependent variables cannot be fully established.

Despite these limitations, this study provides valuable information in support of the literature and has several major strengths. The number of people with type 2 diabetes was large, and to our knowledge larger than any European previously published study of adherence. The studied sample constituted a large nationally representative cohort of diabetic patients. Therefore, the generalizability of the results to countries with similar health care system is high. This study, combining multiple data sources (self-reported, medical-care providers, data from the two main National Health Insurance Systems), provided a large number of diabetes-related variables.

In summary, medication adherence is vital for effective diabetes management. Our findings point towards the interest of fine-tuning the primary care provider's approach to the individual patient by taking into account medication adherence. More evidence to support specific interventions that will be effective in overcoming adherence challenges for diabetes patients is needed. The patients should have a pivotal role in their diabetes management. Therefore, they need to acquire knowledge and skills, but also the ability for behavioural change, which often requires intensive patient-centred health education. In a country with a high level of access to healthcare, our study demonstrated a low level of medication adherence. Better identification of patients with poor adherence, who require a more specific and rigorous patient physician relationship and individualised suitable recommendations, remains essential to obtain better outcomes in type 2 diabetic patients.
